# Human Lung Epithelial Cells Contain *Mycobacterium tuberculosis* in a Late Endosomal Vacuole and Are Efficiently Recognized by CD8^+^ T Cells

**DOI:** 10.1371/journal.pone.0097515

**Published:** 2014-05-14

**Authors:** Melanie J. Harriff, Meghan E. Cansler, Katelynne Gardner Toren, Elizabeth T. Canfield, Stephen Kwak, Marielle C. Gold, David M. Lewinsohn

**Affiliations:** 1 Portland Veterans Administration Medical Center, Portland, Oregon, United States of America; 2 Department of Pulmonary and Critical Care Medicine, Oregon Health & Sciences University, Portland, Oregon, United States of America; 3 Department of Pediatrics, Oregon Health & Sciences University, Portland, Oregon, United States of America; 4 Department of Molecular Microbiology and Immunology, Oregon Health & Sciences University, Portland, Oregon, United States of America; Colorado State University, United States of America

## Abstract

*Mycobacterium tuberculosis* (Mtb) is transmitted via inhalation of aerosolized particles. While alveolar macrophages are thought to play a central role in the acquisition and control of this infection, Mtb also has ample opportunity to interact with the airway epithelium. In this regard, we have recently shown that the upper airways are enriched with a population of non-classical, MR1-restricted, Mtb-reactive CD8^+^ T cells (MAIT cells). Additionally, we have demonstrated that Mtb-infected epithelial cells lining the upper airways are capable of stimulating IFNγ production by MAIT cells. In this study, we demonstrate that airway epithelial cells efficiently stimulate IFNγ release by MAIT cells as well as HLA-B45 and HLA-E restricted T cell clones. Characterization of the intracellular localization of Mtb in epithelial cells indicates that the vacuole occupied by Mtb in epithelial cells is distinct from DC in that it acquires Rab7 molecules and does not retain markers of early endosomes such as Rab5. The Mtb vacuole is also heterogeneous as there is a varying degree of association with Lamp1 and HLA-I. Although the Mtb vacuole shares markers associated with the late endosome, it does not acidify, and the bacteria are able to replicate within the cell. This work demonstrates that Mtb infected lung epithelial cells are surprisingly efficient at stimulating IFNγ release by CD8^+^ T cells.

## Introduction

*Mycobacterium tuberculosis* (Mtb) is a highly successful respiratory pathogen. The World Health Organization estimates that one-third of the world's population is infected with Mtb, with 8.7 million new cases and 1.4 million deaths in 2012 (WHO). Factors such as HIV infection, smoking, and diabetes considerably increase the risk of developing disease after exposure to Mtb, and the emergence of multi-drug resistant strains of Mtb further compounds the world-wide impact [1]. Mtb is transmitted via aerosol delivery of 2-5 micrometer particles containing the bacterium to the alveolus [2]. Although these particles have the opportunity to interact with cells that line the upper airways, most research has focused on the alveolar macrophage and alveolar type II pneumocytes. Abundant data support a model by which Mtb infects alveolar macrophages, where it survives and replicates in an intracellular phagosomal compartment. In this regard, infection of the alveolar macrophage is thought to be the seminal step leading to Mtb dissemination, granuloma formation and the acquisition of TH1-type immunity. While a TH1-type adaptive immune response and granuloma formation is important to control of Mtb, it does not explain many of the clinical outcomes seen following exposure to Mtb. Household contact studies indicate that half of exposed individuals never get infected with Mtb as measured by a positive tuberculin skin test (TST) [3]. Of those individuals that convert to a TST+ skin test, few actually progress to active disease, leading to uncertainty as to whether they are persistently infected or have cleared the infection.

Our understanding of these clinical outcomes following Mtb exposure requires a more complete understanding of both immunologic and non-immunologic events occurring prior to the induction of the adaptive immune response. The human airway contains a variety of both innate and adaptive mechanisms, all of which can contribute to host resistance to infection with Mtb. These mechanisms include mucous, the ability of cilia to clear pathogens, the presence of defensins and other anti-microbial peptides, and the barrier provided by epithelial cells. Prior work has demonstrated that alveolar type II pneumocytes can become infected with Mtb *in vitro* [4]–[6]. Evidence of infection of human lung epithelial cells *in vivo* comes from the work of Hernandez-Pando and Arriaga. In these studies, the authors demonstrate that Mtb DNA can be isolated from non-phagocytic cell tissue, including the bronchial epithelium, from human and mouse lung tissue where there is no evidence of granuloma formation [7], [8]. Additionally, detailed post-mortem analyses reveal that in the infrequent times where Mtb is cultured from infected patients, there are equal odds of finding the bacterium in “normal” lung tissue vs. a granuloma [9]. Both alveolar Type II pneumocytes and airway epithelial cells (AEC) could contribute to early defense following exposure to Mtb through their ability to produce cytokines, chemokines, antimicrobial B-defensins, surfactants, NOS2 and other molecules that either directly kill Mtb or enhance the anti-microbial function of infected macrophages [10]–[12]. Furthermore, infection of epithelial cells could contribute to the early response to Mtb via interactions with adjacent DC as well as T cells. Here, we note the immunologically rich milieu provided by airway resident bronchial associated lymphoid tissue (BALT). In fact, our recent studies show that a population of non-classical, Mtb-reactive CD8^+^ T (MAIT) cells is both highly enriched in human large airways and able to respond to Mtb infection ex vivo [13]. Additional studies in mice and humans demonstrate that response of MAIT cells to pathogen-infected cells is important to early immune control of the pathogen [14], [15]. Together, these studies indicate a potential role for Mtb-infected AEC in directly stimulating the production of cytokines by innate T cells such as MAIT cells.

In this study, we focused on large AEC, as these cells have ample opportunity to interact with Mtb following inhalation. We evaluated the ability of Mtb-infected MHC-II negative large AEC to stimulate Mtb-specific CD8+ T cells following infection with Mtb. Using a human bronchial epithelial cell model (BEAS-2B) as an antigen presenting cell in an IFN-γ Elispot assay, we directly compared the ability of BEAS-2B and human monocyte-derived DC to stimulate Mtb-specific classically and non-classically restricted CD8^+^ T cells. We find that BEAS-2B cells, on a per cell basis, are surprisingly efficient at stimulating CD8^+^ T cells. Furthermore, we find that primary large airway epithelial cells (LAEC) are similarly efficient. This led us to investigate the intracellular localization of Mtb in cultured and primary AEC following infection. We determined that the Mtb vacuole in BEAS-2B and primary LAEC is distinct from the Mtb phagosome in DC. Our findings indicate that despite a relatively inefficient infection, AEC are highly capable of eliciting responses by T cells, supporting the idea that AEC could act as sentinels following exposure to Mtb. Early responses involving the interaction of Mtb, the lung epithelium, and airway resident T cells may contribute to clearance of Mtb in exposed individuals prior to initiation of an adaptive immune response, or may play a role in priming the adaptive immune response.

## Results

### Mtb infected human AEC are efficiently recognized by CD8^+^ T cells

**Figure 1:**
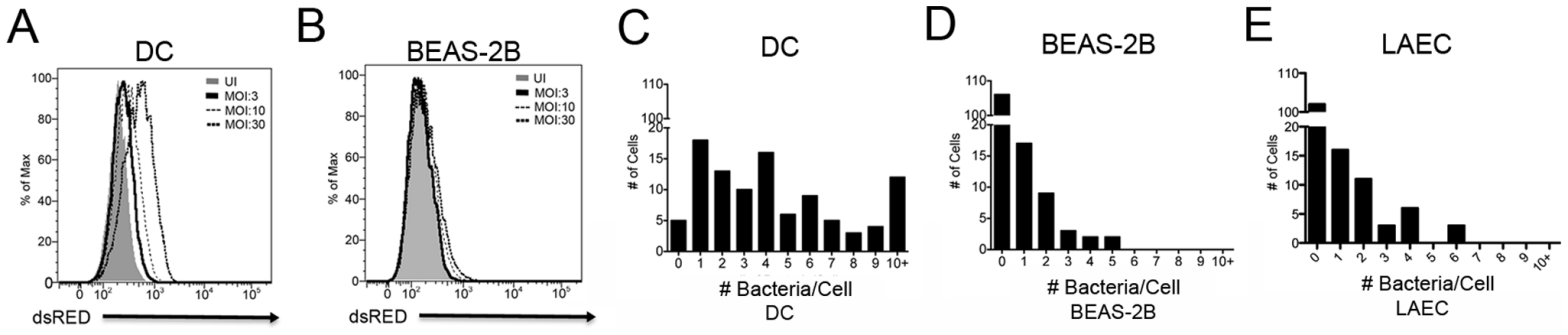
The human lung epithelial cell line BEAS-2B is less efficiently infected with Mtb than human DC. A–B) Primary human DC or the human bronchial epithelial cell line, BEAS-2B, were infected with dsRED Mtb at an MOI of 30, 10, and 3. Fixed cells were assessed for dsRED-H37Rv infection by flow cytometry after 18 hours. FlowJo (TreeStar) was used to calculate the change in geometric mean in BEAS-2B cells or DC for MOI:10. Results are representative of all experiments (N = 3). C–E) DC, BEAS-2B cells, or primary LAEC were infected with Mtb (MOI:7). Infected cells were imaged in an unbiased manner, and the number of bacteria per cell was enumerated. Results are representative of all experiments (N = 3 for DC and BEAS-2B cells, N = 2 for LAEC).

To assess the ability of human AEC to be infected by Mtb, as well as their ability to present mycobacterial antigens to CD8+ T cells, we compared human bronchial epithelial cells to human DC. We initially focused our efforts on the bronchial epithelial cell line, BEAS-2B. To assess the efficiency of infection, DC and BEAS-2B cells were infected for 18 hr with equivalent multiplicities of infection (MOIs) of dsRED-expressing H37Rv Mtb and analyzed by flow cytometry. DC appeared to take up approximately five times the amount of mycobacteria at an MOI:10, as evidenced by the increase in the geometric mean following infection (51.2% increase for DC versus 9.6% increase for BEAS-2B cells, Figure 1a-b). To more accurately compare the percentage of infected cells and the number of bacteria per cell, infected cells, including primary large airway epithelial cells (LAEC) were analyzed using fluorescence microscopy. As expected, following an 18 hr infection with equivalent MOIs (MOI:7), nearly all of the DC (94.1%) were infected by at least one Mtb, while only 24.8% of the BEAS-2B cells and 26.3% of the LAEC were infected. Furthermore, while infected DC often contained multiple mycobacteria, ranging from 1 to 10+ Mtb/cell (Figure 1c), the majority of infected BEAS-2B cells and LAEC contained just 1 or 2 Mtb/cell (Figure 1d-e). BEAS-2B cells were also infected when incubated with a low MOI of Mtb (MOI:1). All results are summarized in Table 1.

**Table 1:** Prevalence and degree of Mtb infection in DC and BEAS-2B cells.

	DC (MOI:7)	BEAS-2B (MOI:7)	LAEC (MOI:7)	DC (MOI:1)	BEAS-2B (MOI:1)
**%Infected Cells**	94.1	24.8	26.3	47.1	8.8
**Average # Bacteria/Cell** (All Cells)	4.8	0.4	0.6	1.0	0.1
**Average # Bacteria/Cell** (Infected Cells)	5.1	1.8	2.4	2.2	1.3

**Figure 2:**
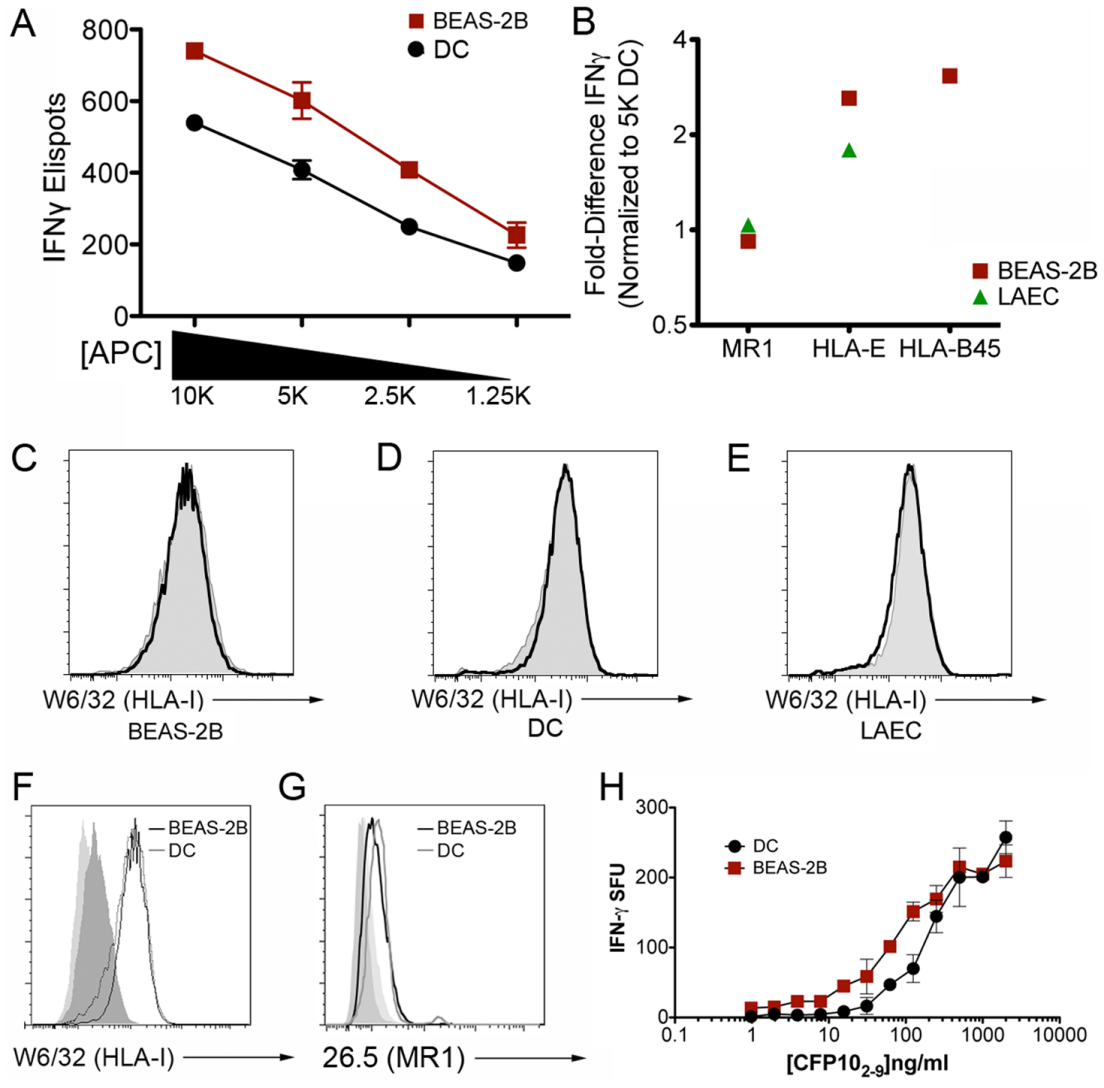
Mtb-infected human AEC are efficiently recognized by Mtb-specific CD8^+^ T cells. A–B) Mtb-infected DC, BEAS-2B cells, or LAEC (MOI:10) were used as APCs in an IFN-γ ELISPOT assay. A) IFN-γ release by the HLA-B45-restricted CD8^+^ T cell clone D466H4 was assessed by ELISPOT assay. 10,000 T cells were used with a titration of APCs from 10,000 - 1,250 cells per well. Results are representative of all experiments (N = 3). Error bars represent the mean and standard error from duplicate wells. B) IFN-γ release by the MR1-restricted (D426B1), HLA-E-restricted (D160 1-23), and HLA-B45-restricted (D466H4) CD8^+^ T cell clones was assessed for Mtb-infected DC, BEAS-2B, or primary LAEC (except D466H4). The fold-difference in IFN-γ release was normalized to the T cell response to Mtb-infected DC (5,000 DC/well) for each APC type and T cell clone. Results are representative of all experiments (N = 3 for DC and BEAS-2B cells and N = 1 for LAEC). C–F) Following fixation in 4% PFA, uninfected or infected BEAS-2B cells, DC, or LAEC were surface stained with either the pan-HLA-I antibody W6/32 (C-F) or the anti-MR1 antibody 26.5 (G) and analyzed by flow cytometry. C-E: shaded histogram, UI; black line, Mtb. F-G: shaded histogram, isotype staining for each cell type. Results are representative of all experiments (N = 5 for W6/32 and N = 4 for 26.5). H) DC or BEAS-2B cells were pulsed with CFP10_2-9_ peptide at the indicated concentrations for 1 hour and assessed for their ability to stimulate IFN-γ production by the HLA-B45 restricted, CFP10_2-9_ specific, D466H4 T-cell clone. APCs were added at 10,000 cells/well, while T cells were kept constant at 500 T cells per well. Results are representative of all experiments (N = 3). Error bars represent the mean and standard error from duplicate wells.

Given the marked difference in the degree and prevalence of Mtb infection in AEC and DC, each cell type was tested for its ability to activate CD8^+^ T cells. A classically restricted CD8^+^ T cell clone (HLA-B45), and non-classically restricted CD8^+^ T cell clones (HLA-E and MR1) were tested for their ability to recognize Mtb-infected AEC and DC. In each case, the AEC or DC were infected for 18 hr at the same MOI with Mtb, then titered from 1×10^4^ ^to^ 1.25×10^3^ cells per well to ensure that the antigen presenting cells (APC) were limiting. IFN-γ production, measured by ELISPOT assay, was used to assess T cell activation. A representative example is shown in Figure 2a for the HLA-B45 restricted T cell clone, D466H4. We note the linear relationship between APC number and T cell activation. In the case of D466H4, Mtb-infected BEAS-2B elicited a stronger T cell response than DC infected at the same MOI. We then normalized the data to T cell activation in response to Mtb-infected DC (value set at “1”). As Figure 2b shows, the D426B1 MR1-restricted T cell clone activation in response to Mtb-infected BEAS-2B cells or primary LAEC was nearly the same as DC. In contrast, activation of the D160 1-23 HLA-E and D466H4 HLA-B45 restricted T cell clones was greater in response to Mtb-infected BEAS-2B or LAEC (for D160 1-23) than observed with DC. D466H4 was not tested with primary LAEC, as the donor used did not express the HLA-B45 allele. These results were surprising based on our observation that BEAS-2B and primary LAEC contain far fewer Mtb after infection with equivalent MOI.

AEC are larger than DC, so one simple explanation for these data is the increased surface area of HLA-I available for T cell interaction. Surface staining for Class I molecules, including HLA-B45 and HLA-E, using the W6/32 antibody indicates that surface Class I expression is similar on both uninfected and infected BEAS-2B cells and DC (Figure 2c–f). Additionally, using the 26.5 anti-MR1 antibody, we observed similar surface expression of MR1 on BEAS-2B cells and DC (Figure 2g). Using peptide exchange as a surrogate for surface Class I expression, we can see that DC and BEAS-2B cells are similar, although at lower peptide concentrations, BEAS-2B retain higher T cell activation compared to DC (Figure 2h). Another possibility is that the Mtb-infected AEC are also making IFN-γ, as shown for the alveolar epithelial cell line, A549 [11]. In our ELISPOT assays, infected AEC are used as controls and do not produce detectable IFN-γ. We performed additional experiments where Mtb-infected BEAS-2B cells were incubated with the D426B1 MR1-restricted T cell clone in a tissue culture plate, and then subsequently analyzed for IFN-γ production by ELISPOT assay and flow cytometry. We see no IFN-γ production by Mtb-infected BEAS-2B cells using either of these methods (data not shown). In agreement with our previously published data with A549s [13], this indicates that the IFN-γ that we are detecting by ELISPOT assay is produced by the T cells. These data demonstrate that despite a lesser degree and prevalence of infection, AEC are efficient at activating CD8^+^ T cells.

### Intracellular infection is required for Mtb-dependent recognition of AEC by CD8^+^ T cells

**Figure 3:**
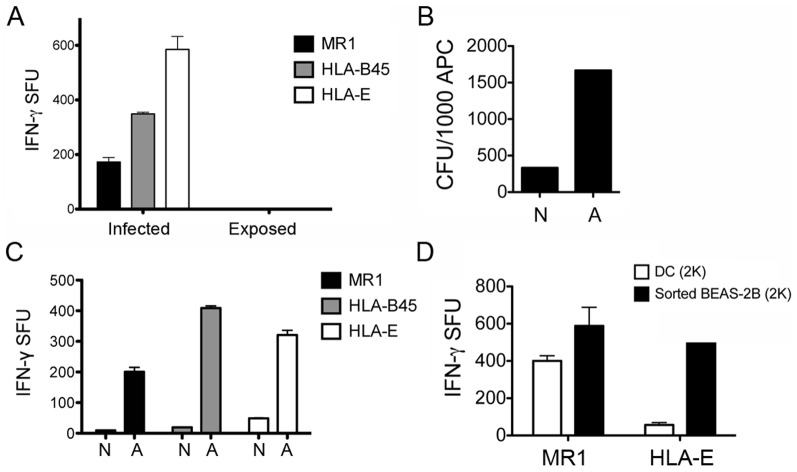
Intracellular Mtb are required for T cell activation. A) BEAS-2B cells were seeded in a 12-well tissue culture plate and a 0.4 uM pore size transwell insert. Cells in the transwell were infected with Mtb (MOI:10) for 18 hrs. Cells from both chambers were then used as APCs (10,000 cells/well) in an IFN-γ Elispot assay. Results are representative of three independent experiments. B-C) Mtb were labeled with streptavidin coated magnetic microbeads prior to infection (MOI:30). A fraction enriched for infected BEAS-2B cells was then obtained by magnetic sorting (adherent). Mtb CFUs were enumerated from each fraction (B) and cells were used as APCs (5,000 cells/well) in an IFN-γ Elispot assay (C). (N = Non-adherent, A = Adherent). Results are representative of three independent experiments. D) The T cell response to the enriched fraction of Mtb-infected BEAS-2B cells (MOI:30, 2,000 cells/well) was compared to an equal number of Mtb-infected DC (MOI:30) in an IFN-γ Elispot assay. Results are representative of two independent experiments. For all assays, error bars represent the mean and standard error from duplicate wells.

Given the relatively inefficient infection of AEC, we considered the possibility that AEC present extracellular antigens. To address the requirement for intracellular infection, a transwell assay was performed. Here, we demonstrated that the infected layer of BEAS-2B cells could elicit T-cell dependent IFN-γ production, whereas uninfected cells exposed to Mtb-infected cells via the transwell were unable to stimulate IFN-γ production (Figure 3a). This result indicated that direct contact of T cells with AEC is required, but did not necessarily demonstrate that intracellular infection was necessary. As a result, we sought to determine whether or not infection with Mtb was a requirement for CD8+ T cell recognition. In this case, we used magnetic-bead sorting to obtain an enriched fraction of Mtb-infected cells. Prior to infection, Mtb were labeled with magnetic microbeads as previously described [16]. After overnight infection, BEAS-2B cells were harvested and sorted by magnetic separation. To determine the effectiveness of the magnetic sorting, quantitative cultures were performed on the sorted populations. As demonstrated in Figure 3b, Mtb was preferentially found in the sorted cell population. Furthermore, when sorted cells were used as APC, the cells enriched for Mtb were enhanced for their ability to stimulate CD8^+^ T cells (Figure 3c). To further demonstrate the efficiency of AEC in activating CD8^+^ T cells, sorted BEAS-2B cells were compared to DC in an ELISPOT assay. As demonstrated in Figure 3d, the sorted BEAS-2B cells elicited a higher response from both the MR1- and HLA-E restricted T cell clones. These results demonstrate that intracellular infection is likely to be required for efficient Mtb-dependent recognition of AEC by CD8^+^ T cells.

### The Mtb vacuole in AEC shares features with the late endosome

Given the unexpected efficiency of antigen processing and presentation, we sought to characterize the intracellular environment of Mtb within the AEC. In addition to control of intracellular pathogens, the makeup of the intracellular compartment is a factor contributing to antigen processing and presentation, [17], [18]. In many cases, a phagosome containing a bacterial pathogen or inert particle will mature rapidly from an early endosome (marked by Rab5) to a late endosome (marked by Rab7), followed by fusion with the lysosome to become an acidified phagolysosome. This hostile environment facilitates the control of intracellular pathogens. In contrast, Mtb is unusual its ability to alter the phagosomal environment. This environment has been extensively characterized in DC and macrophages [19], [20]. In a DC or macrophage Mtb phagosome, the early endosomal marker, Rab5 is present after uptake [21], however subsequent recruitment of EEA1 and Rab7 to the phagosome is blocked [22], leaving the Mtb in a phagosome that shares features with early endosomes. Recent studies indicate that Mtb resides in a Rab7 positive compartment in the alveolar epithelial cell line A549 [6], however little is known about the intracellular localization of Mtb in large airway epithelial cells.

**Figure 4:**
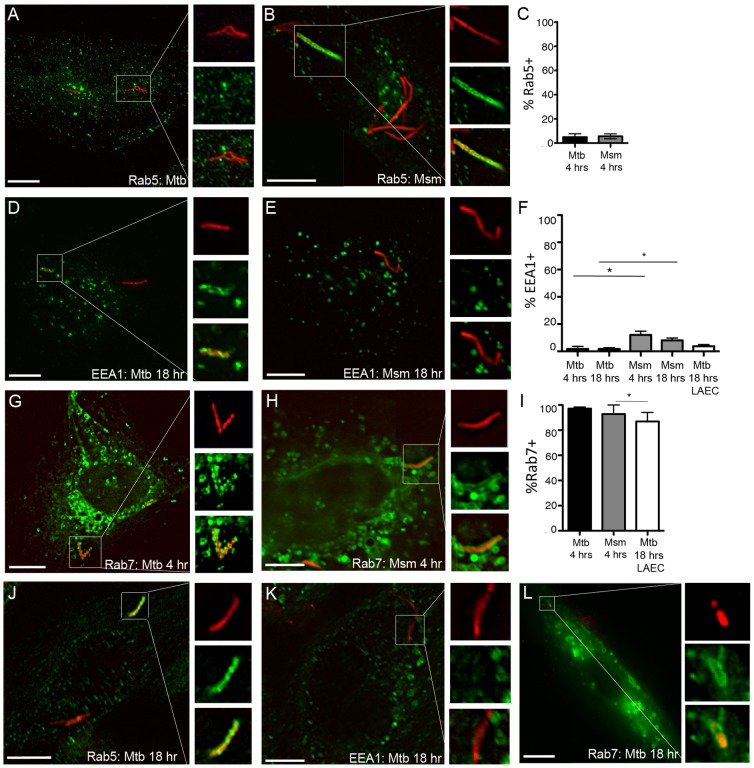
The Mtb vacuole in AEC retains features of a late endosome. A–F) BEAS-2B cells were infected with dsRED-expressing Mtb or RFP-expressing *M. smegmatis* (Msm) at MOI:5 for 4 or 18 hours. Infected cells were fixed and stained for early endosomal markers Rab5 and EEA1. G-I) BEAS-2B cells were transfected with a plasmid expressing a GFP-Rab7 fusion construct for 6 hours prior to infection and fixed following a 4 hour infection. For each condition, at least 200 intracellular bacteria were counted from 3 independent experiments. Mtb-containing vacuoles were categorized as positive or negative, the mean percent positive and standard error were determined, and a Student's t-test was used to determine statistical significance between groups. A,D,G (Mtb); B,E,H (Msm). *p<0.05. J–L) Primary LAEC were infected with dsRED Mtb at MOI:5 for 18 hours and analyzed as described above. N = 2. Scale bar = 10 uM. For all assays, error bars represent the mean and standard error of the mean (SEM) for all events. Images shown are representative of Mtb or Msm that were positively (B,D,G,H,J,L) or negatively (A,C,K) associated with each marker.

**Figure 5:**
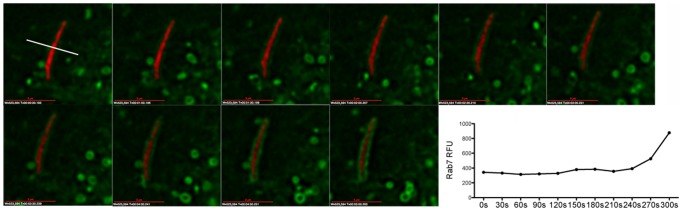
Rab7 accumulates in Msm-containing vacuoles. From 0–2 hours following synchronized infection, individual Rab7-GFP expressing BEAS-2B cells containing RFP-Msm were imaged sequentially. Each cell was imaged every 30 sec for 5 minutes. Shown is a representative 5 minute time-lapse sequence of Rab7 accumulation on an RFP-Msm compartment. In each experiment, we obtained time-lapse images from 8–10 cells. This experiment was repeated four times. The graph represents the GFP relative fluorescent units (RFU) calculated at the perpendicular transection of the bacterium in a single 0.2 uM z-plane at each time point, as shown in the first panel. Scale bar = 3 uM.

To define the intracellular localization of Mtb and its non-pathogenic relative, *M. smegmatis* (Msm) in AEC, we performed a time-course analysis for markers of maturation in Mtb- and Msm-infected cells (MOI:5) using fluorescence microscopy. In contrast to what has been published for the DC Mtb phagosome, very few Mtb (4.9+/−2.7%) or Msm (5.4+/−2.1%) were associated with the early endosomal marker Rab5 (Figure 4a–c), or its effector EEA1 (1.8+/−1.8% for Mtb and 12.1+/−2.7% for Msm) (Figure 4d–f) 4 hours following infection. This did not change for Rab5 (data not shown) or EEA1 (Figure 4f) 18 hours following infection. In contrast, nearly all Mtb and Msm were associated with the late endosomal marker Rab7 by 4 hours following infection (97.1+/−1.2% and 92.9+/−7.1%, respectively) (Figure 4g–i). Although the percentage of EEA1+ Msm vacuoles is significantly higher than observed for Mtb (p<0.05), both Msm and Mtb are significantly more likely to be associated with Rab7 (p<0.001). These findings were confirmed in primary LAEC (Figure 4f,i,j-m). There were significantly fewer Mtb associated with Rab7 in primary LAEC (86.7+/−7.2%) compared with BEAS-2B cells (p<0.05), however there was not a significant difference in Mtb association with EEA1 for BEAS-2B cells or primary LAEC, and as seen with BEAS-2B cells, Mtb were more likely to be associated with Rab7 in LAEC (p<0.001). Using live cell imaging, we observed Rab7 accumulating on the membranes surrounding Msm (Figure 5) at time points from 0–2 hours after infection of BEAS-2B cells. These results lead us to conclude that the Mtb vacuole in AEC is distinct from the DC or macrophage phagosome in that it rapidly acquires features of a late endosome.

**Figure 6:**
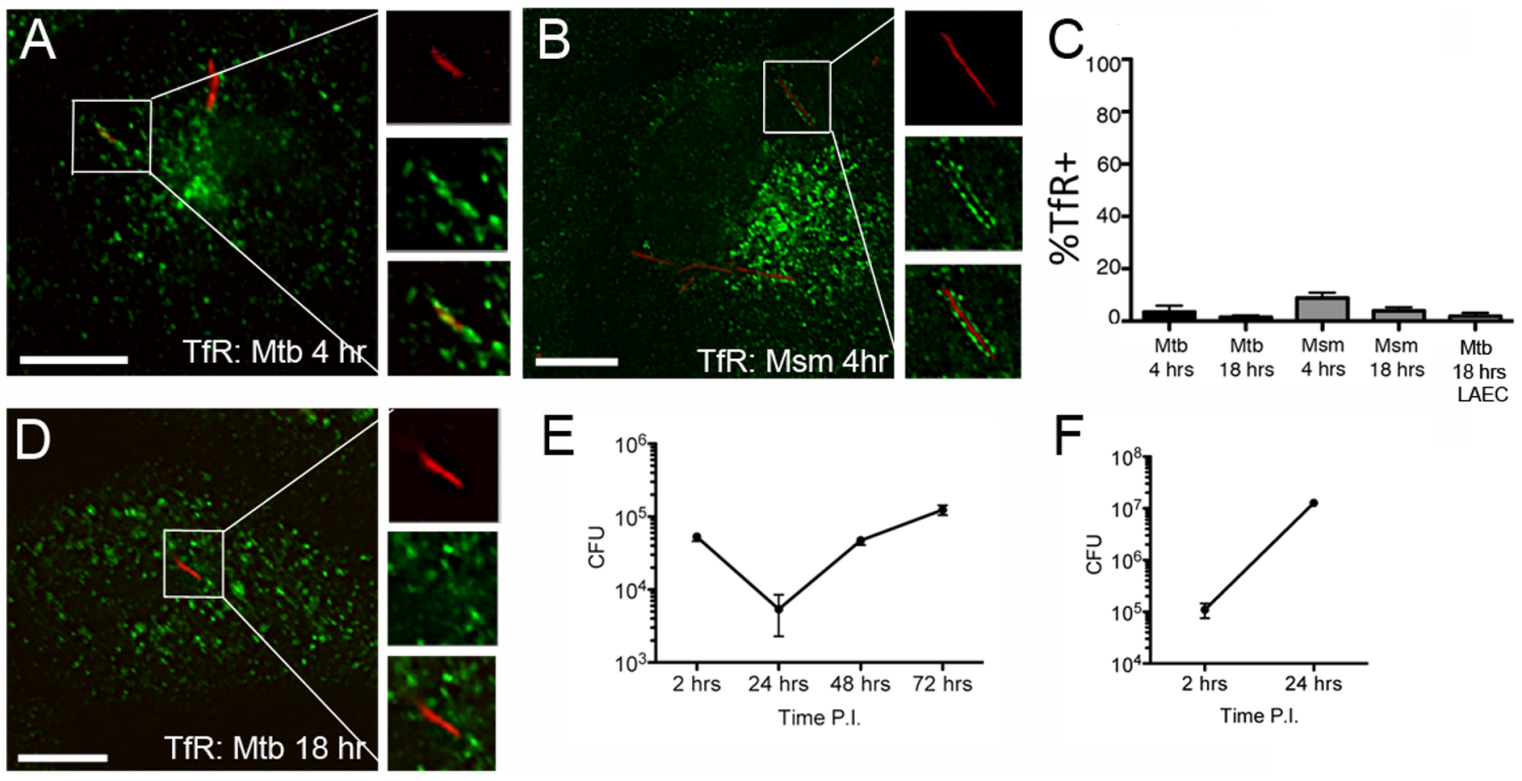
Transferrin receptor (TfR) does not accumulate at high levels in the Mtb vacuole. A–C) BEAS-2B cells were infected with dsRED-expressing Mtb or RFP-expressing Msm at MOI:5 for 4 or 18 hours. Infected cells were fixed and stained for TfR. At least 200 intracellular bacteria were counted from 3 independent experiments. Mtb-containing vacuoles were categorized as positive or negative, the mean percent positive and standard error were determined, and a Student's t-test was used to determine statistical significance between groups. A (Mtb); B (Msm). Images are representative of TfR-positive compartments for Mtb and Msm. Error bars represent the mean and SEM for all events. Scale bar = 10 uM. D) Primary LAEC were infected with dsRED-expressing Mtb at MOI:5 for 18 hours. Infected cells were fixed and stained for TfR and analyzed as described above. Image is representative of a TfR-negative compartment. Scale bar = 10 uM. E-F) BEAS-2B cells were infected with dsRED-Mtb or RFP-Msm (MOI:10). Infection was synchronized by centrifugation. After a 2 hour infection, cells were washed three times with PBS, and wells replenished with media. Cells were harvested and counted, and lysates were plated in triplicate after the 2 hour infection, and at 24, 48, and 72 hours following infection. Error bars represent the mean and SEM from three experiments (Mtb) or the mean and standard error from triplicate wells (Msm).

In the DC and macrophage Mtb phagosome, fusion of recycling endosomes containing transferrin and transferrin receptor (TfR) with the phagosome is promoted through Rab5 dependent mechanisms [23]. Consistent with these findings, we demonstrate that Mtb and Msm vacuoles in AEC also have low levels of TfR. TfR was observed in 3.5+/−2.4% and 8.8+/−2.2% of Mtb and Msm compartments at 4 hrs post infection respectively, with little change at 18 hours (Figure 6a–c). These findings were confirmed in primary LAEC 18 hours after infection (Figure 6c–d). We were unable to detect any Mtb compartments associated with another recycling endosome marker, Rab11 (data not shown). Phagosomal Mtb utilize tranferrin and the transferrin receptor (TfR) to acquire iron from intracellular pools [24], which is required for replication in host cells [25]. We predicted that diminished association with TfR could correlate with impaired mycobacterial replication in AEC. After an initial decrease in the first 24 hours, Mtb replicated approximately one log over the next 48 hour period within BEAS-2B cells (Figure 6e). Intracellular Msm also rapidly replicates in BEAS-2B cells, increasing two logs in the first 24 hours (Figure 6f), leading to cell death between 24 and 48 hours (data not shown). These results indicate that there is sufficient intra-vacuolar iron to support mycobacterial growth.

### The Mtb vacuole in AEC acquires the lysosomal associated protein, Lamp1, but does not acidify

**Figure 7:**
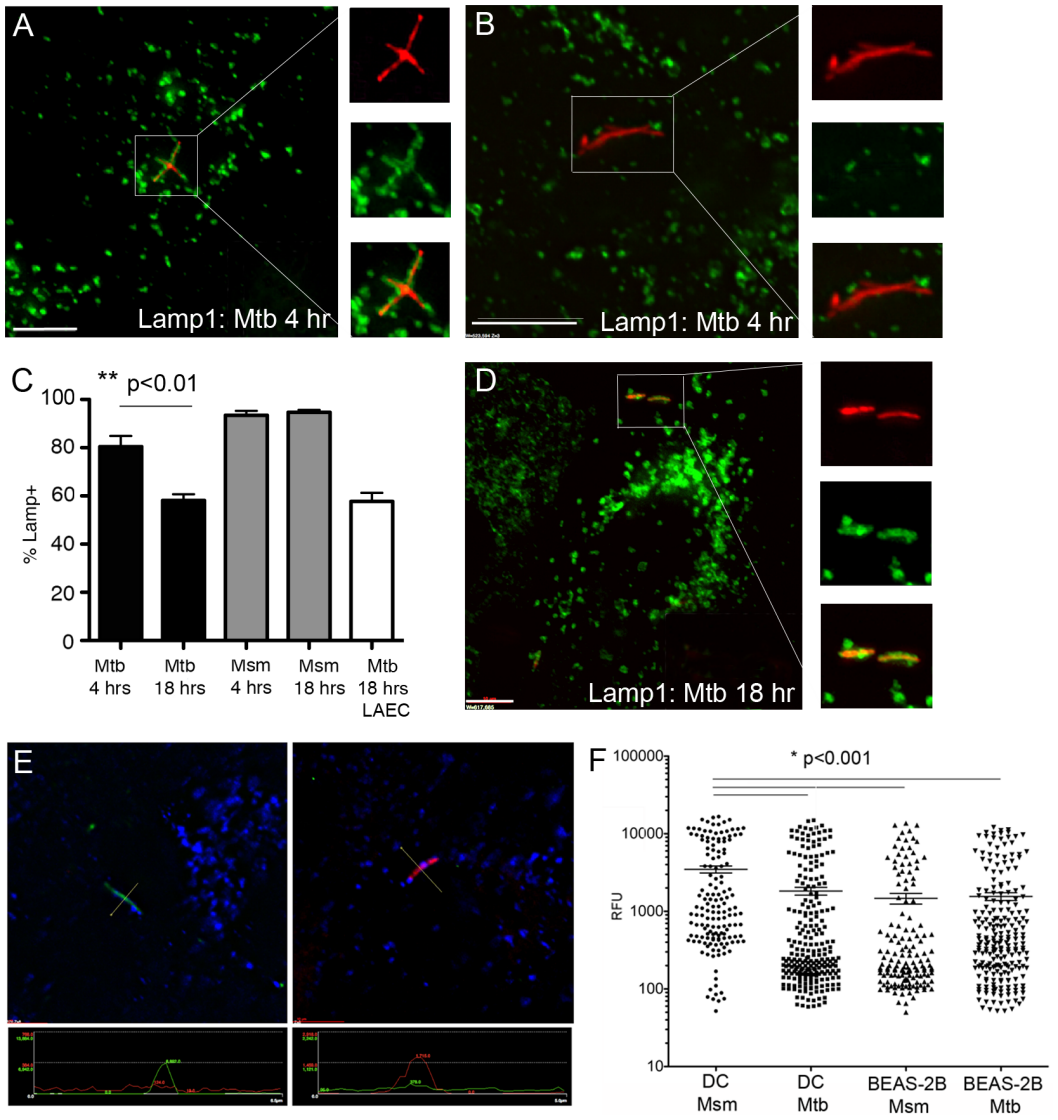
The Mtb vacuole in BEAS-2B cells acquires Lamp1, but does not acidify. A–C) BEAS-2B cells were infected with dsRED-expressing Mtb or RFP-expressing Msm at MOI:5 for 4 or 18 hours. Infected cells were fixed and stained for the lysosomal associated protein, Lamp1. Over 200 intracellular bacteria were counted from 4 independent experiments. Mtb-containing vacuoles were categorized as positive or negative, the mean percent positive and standard error were determined, and a Student's t-test was used to determine statistical significance between groups. Error bars represent the mean and SEM for all events (**p<0.01) D) Primary LAEC were infected with dsRED-expressing Mtb at MOI:5 for 18 hours and analyzed as described above. Scale bar = 10 uM. Images are representative of Lamp1-positive (A,D) or Lamp1-negative (B) Mtb containing compartments. E-F) DC or BEAS-2B cells were infected with GFP-expressing Mtb or GFP-expressing Msm linked to the pH sensitive dye pHrodo Red. Cells were fixed 18 hours after infection and images were acquired on a DeltaVision Core DV wide-field microscope. E) pHrodo signal from perpendicular transection on a single 0.2 µm z-stack was used to generate the relative fluorescence unit (RFU) data point for each individual bacterium. The image on the left is an example of a GFP+ bacterium (green) associated with low pHrodo Red (red) signal, while the image on the right is an example of a bacterium with low GFP signal, but high pHrodo Red signal. Lines on the image indicate the perpendicular transection of the bacterium used to generate the RFU for GFP and pHrodo Red indicated in the panels below the images. F) The pHrodo Red RFU for at least 200 individual bacteria from 4 independent experiments for each condition was plotted on a log scale. Error bars represent the mean RFU and SEM for all events. (*p<0.001)

In DC, although the maturation of the Mtb phagosome is delayed or blocked, the phagosome does acquire the lysosomal associated protein, Lamp1 [16]. Mtb also prevents acidification of the DC phagosome by blocking the acquisition of the V-ATPase transporter [26]. Non-pathogenic mycobacteria such as Msm cannot block this transition, such that the Msm phagosome in DC fuses with the lysosome and acidifies [27]. Given the presence of the late endosomal marker Rab7 on the epithelial cell Mtb and Msm vacuole, we predicted there could be differences in the acquisition of Lamp1 and acidification of the Mtb vacuole in AEC. In BEAS-2B cells, nearly all Msm were associated with Lamp1 at early and late time points, while Lamp1-associated Mtb compartments significantly decreased from 80.5+/−4.4% at 4 hrs following infection to 58.1+/−2.6% at 18 hrs following infection (p<0.01) (Figure 7a–c). These findings were confirmed in primary LAEC 18 hours after infection (57.7+/−3.6% Lamp1 positive Mtb vacuoles) (Figure 7c–d). These results indicate that although mycobacterial DC phagosomes and AEC vacuoles can acquire Lamp1, there is more heterogeneity in Mtb-containing BEAS-2B vacuoles.

To analyze the acidification of the mycobacterial vacuole in BEAS-2B cells, the pH-dependent dye, pHrodo, was linked to Mtb-eGFP or Msm-GFP prior to infection. After 18 hours the cells were fixed and imaged, and the intensity of the pHrodo dye (relative fluorescent units, RFU) was assessed in a single 0.2 uM z-plane for individual phagosomes or vacuoles containing bacteria (Figure 7e). As expected, the average Msm-containing phagosome in DC had significantly higher pHrodo RFU (3484+/1360 vs. 1830+/−209, p<0.001) than the average Mtb-containing phagosome, indicating increased acidification (Figure 7f). In contrast, the pHrodo intensity for Mtb and Msm vacuoles in BEAS-2B cells (1558+/−178 vs. 1473+/−232, respectively) was not significantly different from that of Mtb phagosomes in DC (Figure 7f), indicating that the AEC vacuole does not acidify for Mtb or Msm. In all cases, there was a highly acidic population of phagosomes, possibly representing non-viable organisms (indicated by a correlation to low GFP signal). Interestingly, the high pHrodo intensity vacuoles in BEAS-2B cells were relatively less acidic than the high pHrodo intensity phagosomes in DC. These results demonstrate that the acquisition of late endosome and lysosome markers (Rab7 and Lamp1) in AEC Mtb and Msm vacuoles is not associated with an increase in acidification.

### The Mtb vacuole in AEC contains MHC-I molecules

**Figure 8:**
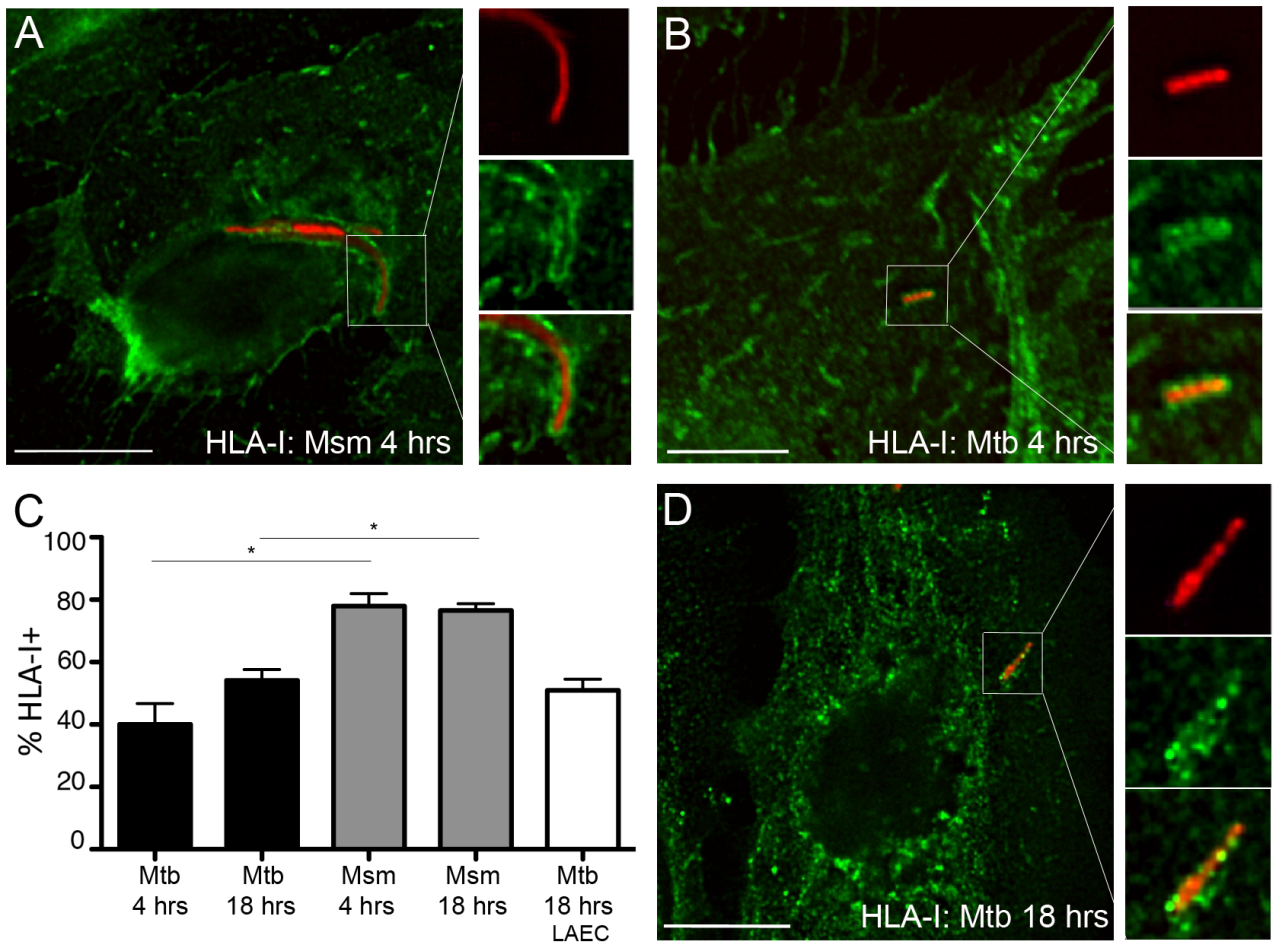
The Mtb vacuole in epithelial cells acquires Class I molecules. A–C) BEAS-2B cells were infected with dsRED-expressing Mtb or RFP-expressing M. smegmatis at MOI:5 for 4 or 18 hours. Infected cells were fixed and stained for Class I molecules (W6/32). At least 200 intracellular bacteria were counted from 3 independent experiments. Mtb-containing vacuoles were categorized as positive or negative, the mean percent positive and standard error were determined, and a Student's t-test was used to determine statistical significance between groups. A (Mtb); B (Msm). Scale bar = 10 uM. D) Primary LAEC were infected with dsRED-expressing Mtb at MOI:5 for 18 hours and analyzed as described above. Infected cells were fixed and stained with W6/32. Scale bar = 10 uM. Images are representative of Mtb or Msm associated with HLA-I. Error bars represent the mean and SEM for all events.

Several non-redundant pathways of antigen processing and presentation have been proposed to explain the mechanisms by which Mtb antigens gain access to the HLA-I pathway (reviewed in [28]). Grotzke *et al*. [16] have provided direct evidence that the Mtb phagosome participates in HLA-I antigen loading. To determine whether the AEC Mtb vacuole is similar to DC in this respect, we looked for the presence of MHC-I molecules. HLA-I could be observed in both Mtb and Msm vacuoles (Figure 8a–b). HLA-I was observed in 40.0+/−6.7% of Mtb-containing vacuoles and 78.0+/−4.0% of Msm vacuoles 4 hours post infection. While the proportion of HLA-I positive Mtb vacuoles increased modestly by 18 hours to 54.2+/−3.5% (p = 0.06), Msm-containing vacuoles continued to have a higher proportion containing HLA-Ia at this later time point (76.5+−2.1%, Figure 8c). Although the intracellular staining pattern for Class I in the primary LAEC was not identical to BEAS-2B cells, Class I was associated with Mtb-containing vacuoles at a similar percentage as was observed for BEAS-2B cells 18 hours after infection (51.0+/−3.5%, Figure 8c–d). These results indicate that Mtb antigens may access HLA-I processing and presentation pathways via the intracellular compartment, as we have observed in DC.

## Discussion

At present, many of the events that occur following exposure to aerosolized Mtb remain poorly defined, and are likely critical in determining who, following exposure, is productively infected and hence at higher risk for disease progression. Although the role of alveolar macrophages in progression of disease is not at question, we note that airway epithelial cells are highly likely to encounter Mtb following aerosol exposure, and may therefore contribute to the initial response to Mtb exposure in a number of ways. AEC may participate in the clearance of Mtb through the action of cilia, as well as the secretion of anti-microbial proteins such as surfactants and defensins. Additionally, alveolar type II pneumocytes are known to produce cytokines, chemokines, antimicrobial molecules, and other innate modulators upon exposure to Mtb that may directly impact the growth of Mtb [10], [12]. AEC express MHC-I molecules, and can directly present intracellular antigen(s) to resident airway CD8^+^ T cells following infection. Effector molecules, including IFN-γ, TNF-α, and granzyme, that are released by T cells following antigen-specific activation could result in direct killing of the infected cell as well as Mtb. Additionally, release of T cell cytokines could enhance the subsequent acquisition of adaptive immunity. For example, IFN-γ might act to facilitate the maturation and subsequent release of IL-12 by lung-resident DC or facilitate the recruitment and maturation of non-resident DC. As a result, we postulate that Mtb infection of AEC could serve as a sentinel event in the host response to Mtb. In this work, we specifically look at the ability of infected AEC to activate CD8^+^ T cells, as well as the intracellular fate of Mtb following infection of AEC. Our previous findings indicate that both classically and non-classically restricted CD8^+^ T cells are able to respond directly *ex vivo* to Mtb-infected epithelial cells lines as well as primary large airway epithelial cells [13]. Here, we show that, despite a low degree and prevalence of infection, Mtb-infected AEC are surprisingly efficient at stimulating IFN-γ release by both classically and non-classically restricted CD8^+^ T cells, supporting a potential role for AEC as sentinels.

One explanation for the surprisingly efficient ability to stimulate CD8+ T cells is that antigen is more abundant in the BEAS-2B cells, on a per cell basis. Clearly, the amount of bacteria per cell was much lower for BEAS-2B cells compared to DC, suggesting this is not the simple explanation. It is also possible that AEC are able to efficiently obtain extracellular antigen. However, transwell and magnetic sorting experiments indicate that intracellular bacteria are the primary source of antigen. Another simple explanation would be that AEC have either an increased number of HLA-I molecules, or that these molecules contain a proportionally higher number of mycobacterially-derived ligands. In this regard, we note that the expression of HLA-I and MR1 on BEAS-2B cells was comparable to DC. However, peptide-loaded BEAS-2B cells were modestly superior to DC in the presentation of peptide. Whether or not this reflects other events associated with T cell activation, and/or formation of the immune synapse is not clear. However, this does not explain the rather dramatic ability of mycobacterially-infected AEC to activate T cells.

In general, presentation of phagosomal antigens on Class I molecules poses a challenge to DC, as phagosomes are a component of the Class II processing pathway. Studies using latex beads have identified several Class I presentation pathways for phagosomal antigens (reviewed in [29]). These include a vacuolar pathway, a conventional cytosol to endoplasmic reticulum pathway, and a cytosol to phagosome pathway, all of which are pathways that have been described for processing and presentation of Mtb antigens. Although processing and presentation of Class I Mtb antigens by the vacuolar pathway has been demonstrated, Grotzke et al. [30] demonstrated that HLA-I Mtb antigens are preferentially processed and presented by cytosolic pathways in DC. In this regard, we speculate that the availability of the HLA-II processing machinery in DC might actually diminish the protein available to enter the HLA-I processing pathway. Although it is still unknown how phagosomal Mtb antigens access the cytosol, cathepsin-dependent degradation of Mtb proteins in the late endosome may reduce the amount of Mtb antigen available for cytosolic processing. AEC do not express MHC-II, and therefore would not have competition for phagosomal antigens, providing a possible explanation for efficient MHC-I loading and subsequent T cell activation. With decreased competition for Class II, loading of Class I molecules in the phagosome could be particularly efficient. In this regard, we have shown that the HLA-E::antigen complex can be found in the DC Mtb phagosome [16]. Given the presence of HLA-I as well as components of the PLC, we have postulated that loading of HLA-I can occur in the phagosome. In this regard, we note that HLA-I is stably present in the AEC Mtb vacuole. As a result, we postulate that enhanced phagosomal processing of Mtb-antigens might also account for the increased T cell activation that we observed.

Alternately, the mechanism by which Mtb infects AEC, and the resulting intracellular localization of the Mtb, could have a direct effect on T cell activation. Studies demonstrate that antigens acquired by cells via different mechanisms, including phagocytosis, endocytosis, pinocytosis, or receptor-mediated uptake, access distinct intracellular vacuoles, and subsequently access different antigen processing pathways for cross-presentation [31], [32]. For example, macrophages that endocytose OVA via the mannose receptor (MR) are capable of activating OT-1 cells. MR-deficient macrophages, despite endocytosing similar levels of OVA via scavenger receptors, are unable to activate OT-1 T cells [31]. We were unable to identify a specific mechanism of infection (i.e. receptor-mediated, macropinocytosis, etc.) by the non-phagocytic BEAS-2B cells (data not shown) for either Mtb or Msm. However, we did look at the resulting intracellular localization of Mtb and Msm in BEAS-2B cells and primary LAEC. In AEC, the Mtb vacuole is heterogeneous and dynamic, but largely retains characteristic markers of a late endosome. This phenotype is distinct from what has been published for the DC Mtb phagosome, suggesting the mechanisms of uptake and vacuole maturation are not the same in AEC and DC. Surprisingly, the Mtb- and Msm-containing vacuoles in BEAS-2B cells were very similar, including having comparable relative pH levels as observed in the Mtb phagosome in DC. In this regard, we speculate that both the virulent Mtb and the non-virulent Msm share the capacity to inhibit phagolysosomal fusion and acidification. Alternately, it is possible that the specific mycobacterial compartment in AEC is not one that would normally acidify. In DC, the slow acidification of the phagosome favors export of protein antigens into the cytosol for digestion by the proteasome and subsequent cross-presentation. The similar pH suggests that the conditions within the AEC vacuole may be ideal for optimal antigen presentation. These similarities and differences may lead us to a mechanism explaining the efficient antigen presentation. Our current studies are directed at identifying the distinct vesicular trafficking pathways involved in Mtb infection of AEC, maturation of the Mtb-containing vacuole, and the subsequent impact on antigen presentation.

In conclusion, we find that after infection with relatively few mycobacteria, AEC are highly efficient at presenting antigen to CD8^+^ T cells. As such, AEC may have properties that allow them to be highly sensitive pathogen recognition cells for both non-classical and classical CD8^+^ T cells. Our data suggest that infection of a single AEC could prove to be a sentinel event in the early recognition and subsequent control of Mtb. These findings have implications both for the understanding of the early immune response to pathogens such as Mtb, as well as for more rational vaccine development.

## Materials and Methods

### Reagents and antibodies

The following antibodies were used for microscopy: α-Lamp1 (H5G11, SCBT), α-HLA-I (W6/32, Serotec), α-Rab5 (Rab5-65, AbCam), α-EEA1 (14/EEA1, BD Biosciences), α-Rab7 (Rab7-117, Sigma), α-TfR (M-A712, BD Biosciences). CFP10_2-9_ peptide was synthesized by Genemed Synthesis, Inc. Pronase-digested cell wall from *M. tuberculosis* was obtained from Colorado State University (Karen Dobos). Rab7-GFP (pEGFP-C1 backbone) and Rab11-GFP (pEGFP-C1 backbone) plasmids were obtained from AddGene. pHrodo succinimidyl ester pH indicator dye was obtained from Molecular Probes.

### Bacteria and cells

The H37Rv strain of *M. tuberculosis* expressing dsRED was a kind gift from Joel Ernst the H37Rv strain of *M. tuberculosis* expressing GFP was a kind gift from David Sherman, and the mc^2^155 strains of *M. smegmatis* (Msm) expressing RFP (Msm-RFP) and GFP (Msm-GFP) were a kind gift from Luis Bermudez. Bacterial strains were grown in Middlebrook 7H9 broth supplemented with Middlebrook ADC (Fisher), 0.05% Tween-80, and 0.5% glycerol. Before infection, bacteria were passaged through a tuberculin syringe 15 times to obtain a single cell suspension, and used for infection at the multiplicity of infection (MOI) indicated for each assay. For CFU counts, bacteria were plated on 7H10 agar supplemented with Middlebrook ADC.

The bronchial epithelial cell line BEAS-2B (CRL-9609) was obtained from ATCC and cultured in DMEM +10% FBS, respectively. PBMCs were isolated from whole blood obtained by apheresis. Monocyte-derived dendritic cells (DC) were prepared as described [33]. Briefly, PBMC were resuspended in 2% heat-inactivated human serum (HuS) in RPMI and allowed to adhere to a T-75 (Costar) flask at 37C for 1 hr. After gentle rocking, non-adherent cells were removed and 10% heat-inactivated HuS in RPMI containing 10 ng/ml IL-4 (Immunex) and 30 ng/ml GM-CSF (Immunex) was added to the adherent cells. After 5 days, cells were harvested with cell-dissociation medium (Sigma-Aldrich) and used as indicated in assays. De-identified lungs were obtained from the Pacific Northwest Transplant Bank (PNTB). Primary large airway lung epithelial cells were derived from the trachea as previously described [13], [34].

CD8+ T cell clones D160 1-23, D466H4, and D426B1 have been previously described [13], [30], [35]. Briefly, D160 1-23 is HLA-E restricted, D466H4 is HLA-B45 restricted, and D426B1 is MR1 restricted. T cell clones were expanded as previously described [36].

### Ethics statement

This study was conducted according to the principles expressed in the Declaration of Helsinki. Study participants, protocols, and consent forms were approved by the Oregon Health & Science University Institutional Review Board (IRB00000186). Written informed consent was obtained from all participants. Uninfected adults were recruited from employees at Oregon Health & Science University as previously described [37]. Uninfected individuals were defined as healthy individuals with a negative tuberculin skin test and no known risk factors for infection with Mtb.

### ELISPOT assays

Day 5 DC were plated in 24-well ultra low adherence (ULA) plates at 5×10^5^/well in RPMI/10% heat-inactivated HuS supplemented with GM-CSF and IL-4. BEAS-2B cells were plated in 6-well plates at 4×10^5^/well in DMEM/10% HS. Cells were infected with dsRED-H37Rv at MOI:10, unless otherwise indicated. For magnetic sorting of Mtb-infected BEAS-2B cells, dsRED-H37Rv was labeled with magnetic microbeads prior to infection as described previously [16], and cells were harvested after 18 hours infection (MOI:30). For magnetic sorting, infected cells were incubated on ice for 5 min on an EasySep magnet. Following two washes, cells that remained adhered to the magnet were collected and used as antigen presenting cells in an IFNγ ELISPOT as previously described [35]. BEAS-2B cells were lysed and plated to enumerate CFUs in the adherent and non-adherent populations. For the experiments shown in Figure 1e, CFP10_2-9_ peptide was added at the indicated concentrations to 10,000 uninfected DC or BEAS-2B cells in the replicate wells of an ELISPOT plate for 1 hour prior to addition of D466H4 T cells. For transwell assays, BEAS-2B cells were plated on a transwell permeable support with 0.4 µm pore size PET membranes (Corning) prior to infection.

### Replication assay

DC or BEAS-2B cells were infected with Mtb or Msm at an MOI of 10∶1 for 2 hours at 37C degrees. Infection was synchronized by centrifugation. After 2 hours, cells were washed 3 times with PBS to remove extracellular bacteria and wells were replenished with fresh media. Cells were harvested and lysed at 2 hrs, 24 hrs, 48 hrs, and 72 hrs after infection, and serial dilutions of lysates were plated to determine CFUs at each time point.

### Detection of intracellular Mtb and surface expression of class I

DC or BEAS-2B cells were infected with dsRED-H37Rv at an MOI of 3, 10, or 30 for 18 hr. Infected and uninfected cells were harvested, fixed with 4% PFA, and subsequently analyzed with a BD FACSCanto II flow cytometer and FACS Diva software (BD). All analyses were performed using FlowJo software (TreeStar). For surface staining experiments, uninfected or infected cells were harvested and surface stained with primary antibodies against Class I (W6/32, 1∶1000) or MR1 (26.5, a kind gift from Ted Hansen, biotinylated, 1∶100) for 40 min on ice in the presence of 2%HuS, 2% goat serum, and 0.5% FBS. After washing, goat anti-mouse IgG2a Alexa-fluor 647 or streptavidin-Alexa-fluor 647 were added for 40 min on ice. Cells were washed and fixed with 1% PFA for 18 hr, then analyzed as described above.

To enumerate intracellular bacteria by microscopy DC, BEAS-2B cells, or primary LAEC were infected in 1.5 mm glass bottom chamber slides (Nunc) with dsRED-H37RV at an MOI of 1 or 7 for 18 hr. Infected cells were washed extensively with PBS, then fixed with 4% PFA for 15 min. Fixed cells were incubated for 40 min with a primary antibody against Class I (W6/32, 1∶1000) in the presence of 0.2% Saponin, 2%HuS, 2% goat serum, and 0.5% FBS. After washing, goat anti-mouse IgG2a Alexa-fluor 488 was added for 40 min. Cells were washed and stored with FluoromountG (Southern Biotech) at 4 deg C until imaging. An unbiased approach using Class I staining was employed to select fields for image acquisition. Images were acquired on a high-resolution wide field CoreDV system (Applied Precision) with a Nikon Coolsnap ES2 HQ. Each image was acquired as Z-stacks in a 1024×1024 format with a 60×1.42 NA Plan Apo N objective. Images were deconvolved with an optical transfer function using an iterative algorithm of 10 iterations, Acquired images were analyzed using Softworx Explorer (Applied Precision) or Imaris (Bitplane) as needed to generate three-dimensional renderings. All cells in acquired fields were enumerated, and intracellular bacteria present in cells were subsequently enumerated. Extracellular bacteria or incomplete cells were excluded from analysis.

### Imaging analysis of the Mtb and Msm vacuole in AEC

BEAS-2B cells or primary LAEC were infected with dsRED-H37Rv, eGFP-H37RV, Msm-RFP, or Msm-GFP at MOI:5 for 4 hr or 18 hr. Infection was synchronized by centrifugation. In some cases, cells were transfected with the Rab7-GFP or Rab11-GFP plasmid 6 hrs or 18 hours prior to infection with Mtb or Msm and fixed prior to imaging. At each time point, wells were washed 3 times with PBS, fixed with 4% paraformaldehyde (PFA) for 15 min, washed, and stained with primary antibodies for 40 min in the presence of 0.2% saponin, 2% HuS, 2% goat serum, and 0.5% FBS. After washing, goat anti-mouse IgG1 or IgG2a Alexa-fluor 488, 568, or 647 were added (1∶1000) and incubated for 40 min. Cells were then washed and images were acquired as described above, except that fields were selected in an unbiased approach using the Mtb or Msm. In BEAS-2B cells, for each marker and time point, at least 200 individual bacteria from three independent experiments were combined and counted and analyzed on Softworx Explorer (Applied Precision) or Imaris (Bitplane). For primary LAEC experiments, images were acquired of infected cells from 2-4 donors for each marker. For live cell imaging, BEAS-2B cells were transfected with Rab7-GFP for 12 hours, then infected with Msm-RFP at MOI:10. Infection was synchronized by centrifugation. Cells were kept at 37 deg C and 5% CO_2_ and individual cells were imaged sequentially between 0 and 2 hours after infection. For each cell, time lapse images were acquired every 30 sec for 5 min. The relative fluorescent units (RFU) for GFP signal over time were calculated by transecting the bacterium perpendicularly at a single 0.2 uM z-plane at each time point using Softworx Explorer (Applied Precision) and recording the relative units of fluorescence.

### pHrodo assay for acidification of vacuoles

Prior to infection, eGFP-H37Rv or Msm-GFP were incubated with pHrodo Red succinimidyl ester for 30 min in PBS+0.05% Tween-80. The reaction was stopped with an equal volume 0.4 M Lysine, and bacteria were washed 2 times with PBS+0.05% Tween-80. Labeled bacteria were then used to infect BEAS-2B cells or DC at an MOI of 1. After 18 hours, cells were washed with PBS, fixed with 4% PFA, and stained with α-Lamp1 as described above. Images were acquired on the high-resolution wide field Core DV system (Applied Precision) with fixed excitation conditions. Each individual membrane-bound bacterium was analyzed for relative GFP, pHrodo Red, and Alexa647 (Lamp1) fluorescence units in a single 0.2 uM Z-stack by transecting the bacterium perpendicularly at a single 0.2 uM z-plane using Softworx Explorer (Applied Precision) and recording the relative units of fluorescence for GFP, pHrodo Red, and Alexa647 (Lamp1) along that plane.

### Statistical analysis

Statistical significance was determined using Student's two-tailed t test, unless otherwise indicated.
